# Partial complementation of a DNA ligase I deficiency by DNA ligase III and its impact on cell survival and telomere stability in mammalian cells

**DOI:** 10.1007/s00018-012-0975-8

**Published:** 2012-03-30

**Authors:** Catherine Le Chalony, Françoise Hoffschir, Laurent R. Gauthier, Julia Gross, Denis S. Biard, François D. Boussin, Vincent Pennaneach

**Affiliations:** 1 Equipe Avenir, INSERM, 92265 Fontenay-aux-Roses, France; 2 Laboratoire Génétique de la Stabilité des Chromosomes, Institut de Radiobiologie Cellulaire et Moléculaire, CEA, 92265 Fontenay-aux-Roses, France; 3 Laboratoire de Radiopathologie, Institut de Radiobiologie Cellulaire et Moléculaire, CEA, 92265 Fontenay-aux-Roses, France; 4 UMR 967, INSERM, 92265 Fontenay-aux-Roses, France; 5 Univ Paris Diderot, Sorbonne Paris cité, Fontenay-aux-Roses France; 6 Université Paris XI, Fontenay-aux-Roses, France; 7 Commissariat à l’Energie Atomique, Institut des maladies émergentes et des thérapies nouvelles, Service d’Etude des prions et des Infections Atypiques, 92265 Fontenay-aux-Roses, France; 8 Present Address: Institut Curie, Centre de Recherche, Inserm U612, Bât. 112, Centre Universitaire, 91405 Orsay Cedex, France

**Keywords:** DNA ligase I, DNA ligase III-XRCC1, Replication, Telomeres, Sister chromatid exchanges, Telomere sister fusions

## Abstract

DNA ligase I (LigI) plays a central role in the joining of strand interruptions during replication and repair. In our current study, we provide evidence that DNA ligase III (LigIII) and XRCC1, which form a complex that functions in single-strand break repair, are required for the proliferation of mammalian LigI-depleted cells. We show from our data that in cells with either dysfunctional LigI activity or depleted of this enzyme, both LigIII and XRCC1 are retained on the chromatin and accumulate at replication foci. We also demonstrate that the LigI and LigIII proteins cooperate to inhibit sister chromatid exchanges but that only LigI prevents telomere sister fusions. Taken together, these results suggest that in cells with dysfunctional LigI, LigIII contributes to the ligation of replication intermediates but not to the prevention of telomeric instability.

**Electronic supplementary material:**

The online version of this article (doi:10.1007/s00018-012-0975-8) contains supplementary material, which is available to authorized users.

## Introduction

Rapid and appropriate rejoining of single-strand breaks (SSBs) in the DNA duplex is critical to the preservation of genomic integrity [1–7]. The repair of DNA SSBs involves lesion detection, processing, gap filling, and ligation (for reviews see [8–10]). Single-strand break junction defects in patients are associated with the development of neurodegenerative syndromes [11–13] and cancer predisposition [3]. Patients with DNA ligation defects show greater sensitivity to DNA-damaging agents and also immunodeficiency [6]. Single-strand discontinuity in the DNA backbone is formed at the replication fork during lagging strand DNA synthesis and is transiently introduced by the action of topoisomerases during replication or transcription to release DNA topological constraints. In addition, SSBs are one of the intermediates of several DNA repair pathways that require nucleotide replacement and are also formed directly following exposure to ionizing radiation.

In eukaryotic cells, the sealing of SSBs requires the formation of covalent phosphodiester bonds between adjacent 5′ phosphoryl and 3′ hydroxyl groups in the DNA, a reaction catalyzed by DNA ligases I and III (LigI and LigIII). Both ligases contain a conserved catalytic domain flanked by distinct N- and C-terminal regions, which probably confer some functional specificity [14, 15]. LigaseI interacts with its partner PCNA via the PCNA-binding motif located in its N-terminus. This interaction directs the end-joining activity of LigI to Okazaki fragment junctions on the lagging strand during S phase [16]. In addition, LigI activity is also involved in DNA joining in the long patch sub-pathway during base excision repair (BER) [17] and during double-strand break (DSB) repair by homologous recombination [18]. The LigIII/XRCC1 complex is a component of the SSB repair and short-patch BER sub-pathway [8–10]. In both pathways, the LigIII and XRCC1 interactions that occur through the C-terminal BRCT domains present on both proteins contribute to LigIII recruitment [19]. XRCC1-independent functions for LigIII during alternative non-homologous end-joining (NHEJ) and in the maintenance of mitochondrial integrity have also been recently described [20–22].

To date, only one patient (mutation 46Br) has been identified with a reduction in LigI activity. This arose from a missense mutation in the *LIGI* gene that caused an arginine to tryptophan substitution at amino acid 771 [23]. Expression of the LigI 771Trp mutant in human cells [23–25] or the equivalent mutant in mouse [26] has been found to be associated with a delay in the maturation of newly synthesized Okazaki fragments and a higher rate of sister chromatid exchange (SCE) compared to wild-type LigI cells [5, 6, 26, 27]. LigaseI function has been further shown to be essential in yeast [28], mammalian cells [29], and also during the early stages of animal development [5, 30]. Nevertheless, mouse embryonic fibroblast cell lines derived from *LIGI* ^−/−^ animals, although viable, proliferate with an increased doubling time compared to *LIGI* ^+/+^ cells [5, 6, 26, 30]. In addition, because 46Br human cells and *LIGI* ^−/−^ mouse cells are hypersensitive to 3-amino benzamide [5, 31], a poly (ADP-ribose) polymerase 1 (PARP-1) inhibitor, and as PARP-1 plays a role in the recruitment of the LigIII/XRCC1 complex to SSBs, it has been proposed that LigIII may partially compensate for the LigI defect in proliferating 46Br cells.

We show here that LigIII and its partner protein XRCC1 are required to promote the viability of mammalian LigI-deficient cells and that LigI and LigIII have overlapping functions in the maintenance of genome integrity. Interestingly, whereas LigI and LigIII cooperate to inhibit SCE, the depletion of LigI but not LigIII induces sister telomere fusion, providing evidence for the importance of LigI for telomere stability.

## Materials and methods

### Cell lines and shRNA-depleted cell populations

The mouse embryonic fibroblast cell lines derived from *LIGI* ^+/+^ and *LIGI* ^−/−^ animals were obtained from Dr. Melton [5, 30]. The human simian virus 40-transformed fibroblastic lines 46Br.1G1 (European Collection of Cell Culture) derived from a patient with a LigI deficiency syndrome [23] and AS3WT2 cells derived from a healthy donor [32] were maintained in monolayer cultures in Dulbecco’s modified Eagle’s medium (DMEM) supplemented with 15 % fetal bovine serum, and 20 % Medium 199 (Invitrogen). The HeLa, MCF7, U2OS, and HeLa H2B-GFP [33] lines were maintained in DMEM supplemented with 10 % fetal bovine serum. The culture media for all cell lines contained 200 U/ml penicillin, 200 μg/ml streptomycin, 1 mM sodium pyruvate, and 4 mM l-glutamine. To obtain cell populations in which specific genes were suppressed, HeLa and HeLa H2B-GFP cells were transfected with pEBV vectors expressing an shRNA that targeted *LIGI*, *LIGIII*, *XRCC1*, or *PARP1* [34]. The transfection reagent used was Lipofectamine 2000 (Invitrogen) and cells were selected in standard media supplemented with 250 μg/ml hygromycin B (Invitrogen). When indicated, MCF7, U2OS, and HeLa cells were infected for 24 h by lentiviral particles encoding *LIGI* shRNA. Transduced cells were selected for 3 days in media supplemented with puromycin at a final concentration of 1.5 μg/ml. For dual shRNA expression, HeLa cells previously transfected with the indicated pEBV vectors, were infected by lentiviral particles encoding *LIGI* shRNA.

### shRNA plasmids

shRNA sequences were designed as previously described [34]. Two sequences targeting *LIGI* (NM_000234) at nucleotides 794–812 and 2,693–2,711 were used and showed an identical depletion efficiency. The target sequences were nucleotides 164–182 for *LIGIII* (NM_13975), 2,068–2,086 for *PARP1* (NM_001618) and 1,832–1,850 for *XRCC1* (NM_006297) as previously characterized [34, 35]. An shRNA sequence targeting the Luciferase2 gene at the coding sequence 5′-CCTACGCCGAGTACTTCGA-3′ was used as a control. The double-stranded oligonucleotides were cloned in front of the H1 promoter in the pEBV vector carrying a hygromycin B resistance cassette.

The lentiviral plasmid pTRIPΔ3U-MND-EGFP-PURO was constructed by replacing the *Nco*I-EGFP-*Kpn*I fragment from the pTRIPΔ3U-MND-EGFP plasmid [36] with the *Nco*I-EGFP-puromycin-*Kpn*I fragment from the pEGFP-puromycin plasmid [37]. This conferred enhanced green fluorescence expression and puromycin resistance. The H1-shRNA fragments amplified from pEBV-H1-shRNA by PCR were cloned into the pTRIPΔ3U-MND-EGFP-puromycin plasmid. Lentiviral particles were produced as previously described [36].

### Colony formation assay

Cell populations expressing an shRNA from the pEBV episomal plasmid were trypsinized, counted, and plated at a low density to facilitate the formation of isolated colonies. For the HeLa cell populations expressing two shRNAs, the cells were first transfected with episomal plasmids expressing either a control shRNA, or *LIGIII* or *XRCC1* or *PARP1* targeting shRNAs. After 7 days of selection with hygromycin, the HeLa^CTL^, LigIII^KD^, XRCC1^KD^, and PARP1^KD^ populations were transduced for 24 h with lentiviral particles to introduce either the control or *LIGI* shRNA. The transduced cells were trypsinized, counted, and plated and 24 h later were placed under a combined puromycin and hygromycin selection. For single or double shRNA expression, the colonies were fixed after 10–15 days, and stained with Coomassie blue. Colonies of more than 50 cells were counted.

### Preparation of cell lysates and Western blotting

Triton-insoluble fractions were isolated from cells as previously described [38] with slight modifications. Briefly, adherent cells from a 60-mm dish were rinsed in cold PBS and incubated for 10 min on ice with gentle shaking in 2 ml of buffer A (100 mM NaCl, 300 mM sucrose, 3 mM MgCl2, 10 mM Pipes pH 6.8, 1 mM EGTA, Triton X-100) supplemented with protease inhibitors cocktail 1 (Sigma-Aldrich). The Triton concentration used is indicated in Fig. 3. After the removal of buffer A and rinsing in PBS, adhering cellular material (Triton-insoluble fraction) was harvested by scraping the cells off the dishes into PBS. The pellet was resuspended in 60 μl buffer B (50 mM Tris–HCl, pH 7.5, 20 mM NaCl, 0.1 % SDS) containing 25 U of the DNase Benzonase (Novagen) and protease inhibitors cocktail 1 (Sigma) and incubated at room temperature for 10 min. For the preparation of total cell extracts, the cells were rinsed in cold PBS and harvested by scraping into PBS. The cell pellets were resuspended in 120 μl of buffer B. Laemmli buffer was added to these extracts for immunoblotting analysis with the following primary mouse antibodies: anti-LigI (5H5) (MBL), anti-LigIII (IF3) (GenTex, Inc.), anti-XRCC1 (33-2-5) (Abcam), PARP-1 (a gift from Dr. V. Schreiber), anti-Actin (C4) (Lab Vision CO), and anti-HP1α (1G9) (Euromedex).

### Immunofluorescence microscopy

For the immunofluorescent staining of LigIII or XRCC1 in S phase cells, cells grown on glass coverslips were incubated in 10 μM BrdU (Becton-Dickinson) for 30 min. The cells were then fixed in 4 % paraformaldehyde for 5 min at room temperature (RT), rinsed in PBS, and incubated with 100 % methanol for 10 min at −20 °C. The fixed cells were rinsed once in buffer C [100 mM Tris–HCl (pH 7.5), 400 mM NaCl, 0.2 % Triton X-100 and protease inhibitors] for 10 min at RT and three times in TNT buffer [100 mM Tris–HCl (pH 7.5), 150 mM NaCl, 0.05 % Tween 20]. Cells were then incubated with PBS complemented with 2 % bovine serum albumin fraction V (BSA) (Sigma-Aldrich) for 30 min at RT, prior to incubation with anti-LigIII (BD Biosciences), or anti-XRCC1 (clone 33-2-5) (Abcam) antibodies at a 1:250 or 1:100 dilution, respectively, in 2 % BSA-PBS for 1 h at RT. This was followed by incubation with an anti-mouse antibody coupled to Alexa Fluor 594 (Molecular Probes). For BrdU staining, the DNA was denatured by incubating the cells in 4 N HCl for 20 min at RT and the staining was performed with fluorescein isothiocyanate (FITC)-conjugated anti-bromodeoxyuridine (BrdU) (Becton-Dickinson) in accordance with the manufacturer’s recommendations. For PCNA staining, the cells were incubated with FITC-conjugated anti-PCNA (clone PC10; Chemicon International) at a 1:100 dilution in 2 % BSA-PBS. For the analysis of LigI and PCNA, or for BrdU costaining, cells were fixed with cold methanol and incubated in anti-LigI (clone 5H5; MBL) antibodies at a 1:1,000 dilution in 2 % BSA-PBS. Cells were processed as described above for BrdU or PCNA detection. Nuclear DNA was counterstained with 1 μg/ml 4′,6′-diamidino-2-phenylindole (DAPI). Coverslips were mounted in Dako Fluorescent Mounting Medium. Image acquisition was performed with a Leica confocal microscope SPE (Wetzlar, Germany), using an ACS APO 63.0 × 1.30 oil lens or a Leica DM5500 equipped with a CoolSNAP HQ CCD camera using an HCX PL S—APO 63/1.30 oil lens. The images from mid- and late-S phase cells were processed with ImageJ software (http://rsbweb.nih.gov/ij/). The images from each channel were binarized and we applied a threshold filter in order to remove the background. The images were processed with a colocalization threshold filter to obtain a merge binary image from which non-overlapping pixels were removed. The resulting black-and-white images (labeled thresholded images) were used to determine the percentage of nuclei with more than two late-replication structures (ring- or horseshoe-shape staining). For each double-staining combination, more than 40 nuclei were analyzed per cell line.

### Sister chromatid exchanges (SCE)

To analyze SCE, cells were cultured in complete medium supplemented with 10 µM BrdU for two cell cycles. After the addition of colchicine at 10 µg/ml for 2 h, the cells were collected and incubated in KCl (0.075 M) and human serum (1:6 v/v) for 20 min at 37 °C, fixed in ethanol/acetic acid (3:1 v/v), and spread on cold and clean slides. The slides were incubated in Hoechst (33258) dye at 50 µg/ml for 20 min and then denatured by exposure to ultraviolet (UV) light at 365 nm (Fisher Bioblock Scientific) for 30 min in the presence of 2 × SSC. After washing in bi-distilled water, the slides were further stained with 1.5 % Giemsa and 1.5 % phosphate buffer in bi-distilled water. Metaphases were observed under a microscope (Olympus AX70) and analyzed using the Cytovision system. The SCE frequency was calculated as the total number of SCEs divided by the total number of chromosomes.

### Telomere-fluorescent in situ hybridization (Telo-FISH)

Telomere-fluorescent in situ hybridization was performed using a Cy-3-labeled (CCCTAA)_3_ PNA probe (Applied Biosystems) which is specific for the G-rich telomeric strand. Labeling of metaphase spreads was carried out as previously described [32]. The chromosome preparations were then counterstained with DAPI and observed under a fluorescence microscope (Olympus AX70). Digital images were recorded using a camera JAI and analyzed with the Cytovision system. Sister telomere fusions in at least 35 metaphases were scored and statistical analyses were performed with StatView software.

### Chromosome orientation-FISH (CO-FISH)

Chromosome orientation-FISH was performed as previously described [32, 39–41]. Briefly, cells were cultured in complete medium supplemented with 10 μM BrdU for one cell cycle. Metaphase spreads obtained as described above were stained with Hoechst 33258 and then exposed to UV light and digested with exonuclease III (Promega) to remove newly synthesized DNA strands. Successive hybridizations with FITC-labeled (TTAGGG)_3_ PNA probe (Applied Biosystems), then with a Cy-3-labeled (CCCTAA)_3_ PNA probe allowed the detection, by fluorescence microcopy, of parental telomere strains whereby telomeric lagging strands are stained in red and telomeric leading strands are stained in green.

### Live videomicroscopy

Cells were grown on poly-d-lysine coated glass coverslips. Prior to acquisition, glass coverslips were mounted in a Ludin Chamber (Life Imaging Services). Live microscopy was then carried out using an inverted microscope (Olympus IX81) placed in an incubator chamber (Life Imaging Services) maintained at 37 °C, and coupled with a CoolSNAP HQ camera (Princeton Instruments) controlled by Metamorph software (Universal Imaging) as previously described [41]. The fluorescent GFP images were captured on 10–20 fields using a 20× objective (LCPFI, NA 0.40, Olympus) every 2 min for 10 h. Movies were processed with Metamorph software.

## Results

### DNA ligase III and XRCC1 localize at replication foci in DNA ligase I-deficient cells

**Fig. 1:**
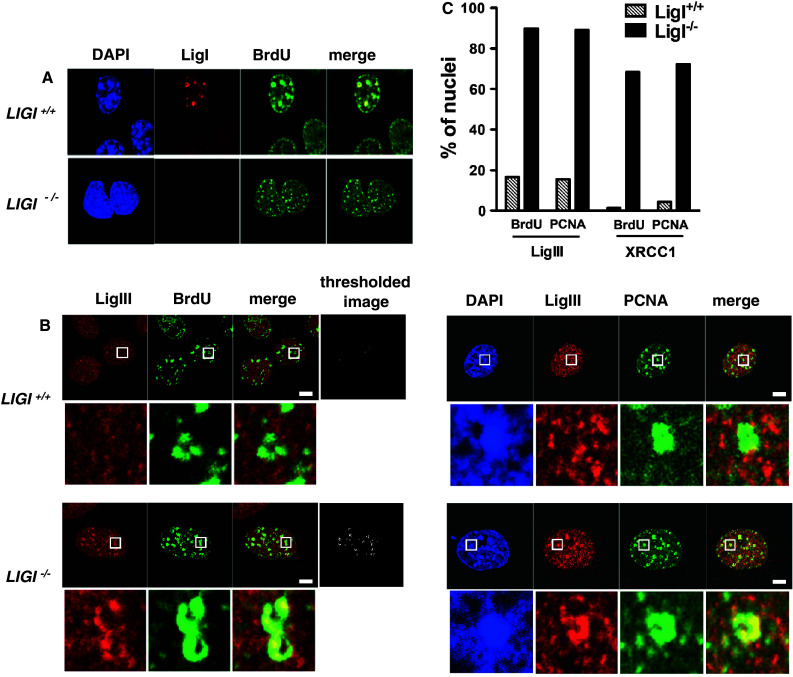
Mouse LigI-deficient cells show endogenous LigIII and XRCC1 accumulation at PCNA- and BrdU-stained foci. **a** Endogenous LigI (*red*) was co-immunodetected with BrdU incorporation sites (*green*) in proliferating wild-type (*LIGI* ^+/+^) and *LIGI* knockout (*LIGI* ^−/−^) mouse cells. **b** Co-immunodetection of endogenous LigIII (*red*) and BrdU incorporation site or PCNA staining (*green*) in late-S phase *LIGI* ^+/+^ and *LIGI* ^−/−^ cells. LigIII signal overlapping with BrdU or PCNA signal is observed in *LIGI* ^−/−^ cells with typical late-S phase BrdU or PCNA staining (enlarged images below showing examples of late-replication structures ring-shape or horseshoe staining). The thresholded images were obtained as described in “Materials and methods”. *Scale bars* 5 μm. **c** Percentages of nuclei with more than two late-replication structures in the depleted cells. For each double staining combination, more than 40 nuclei were analyzed per cell line. Representative images from cells co-immunostained for endogenous XRCC1 and BrdU or XRCC1 and PCNA are presented in ESM Fig. 1b, c

We investigated the nuclear distribution of endogenous LigI, LigIII, and XRCC1 proteins in the nuclei of *LIGI* ^+/+^ and *LIGI* ^−/−^ mouse embryonic fibroblasts cell lines. The S phase cells were identified through the characteristic immunostaining patterns of either incorporated BrdU [42, 43] or chromatin-bound PCNA foci [44, 45]. The BrdU- or PCNA-positive nuclei were categorized into early, mid-, or late-S subgroups based on the characteristic replication patterns. Early S phase nuclei are characterized by a pattern of high density of small BrdU or chromatin-bound PCNA foci spread throughout the nucleus, and mid- or late-S phase mouse cells have a typical ring-shape BrdU and PCNA staining around heterochromatin domains [43]. As expected, endogenous LigI protein was not detectable in *LIGI* ^−/−^ cells and co-localized at BrdU stained DNA replication foci in *LIGI* ^+/+^ cells (Fig. 1a) [46]. The immunostaining of endogenous LigIII protein in the nuclei of non-S phase and early S phase *LIGI* ^+/+^ cells appeared to be evenly distributed with no obvious co-localization with BrdU or PCNA foci (electronic supplementary material, ESM Fig. 1a). However, within the mid- or late-S-phase *LIGI* ^−/−^ population, we could easily observe distinct and intense LigIII (Fig. 1b) and XRCC1 (Fig. 1 and ESM Fig. 1b) foci in a mostly diffuse staining pattern, these foci strongly co-localizing to mid- or late-S-phase ring-shaped BrdU or PCNA foci. Merged images from mid- or late-S-phase cells co-immunostained for BrdU and either LigIII (Fig. 1b) or XRCC1 (ESM Fig. 1b), or from cells co-immunostained for LigIII or XRCC1 and PCNA (Fig. 1b and ESM Fig. 1c) were thresholded. The resulting thresholded images were then used to determine the percentage of nuclei with more than one co-stained mid- or late-S-phase ring-shaped focus under each set of experimental conditions. The percentage of cells with mid- and late-S-phase replication foci co-stained for LigIII/BrdU or LigIII/PCNA increased, respectively, from 16.6 to 15 % in mouse *LIGI* ^+/+^ cells to 89.7 and 89 % in *LIGI* ^−/−^ cells (Fig. 1c). In addition, the percentage of cells with mid- or late-S-phase replication foci co-stained for XRCC1/BrdU or XRCC1/PCNA (Fig. 1c and ESM Fig. 1b, and c), significantly increased from 1.3 to 4.3 % in the *LIGI* ^+/+^ cells to 68.5 and 72.3 % in *LIGI* ^−/−^ cells (Fig. 1c). These observations suggest that a complex containing the LigIII/XRCC1 DNA repair proteins accumulates at mid- and late-S-phase DNA replication sites in LigI-deficient proliferating mouse cells.

**Fig. 2:**
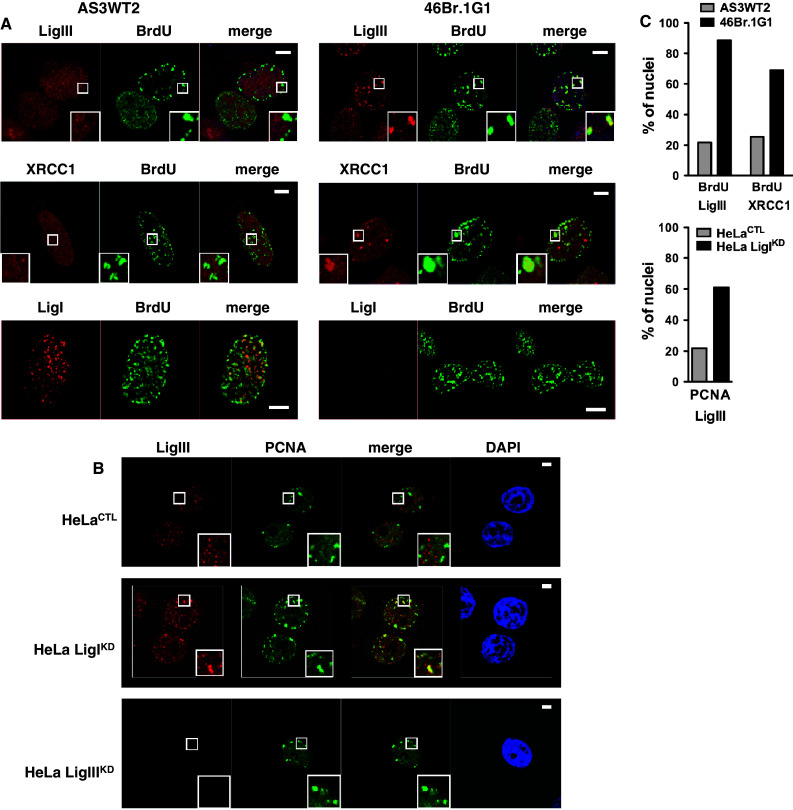
LigIII and XRCC1 proteins accumulate at PCNA and BrdU-stained foci in the 46Br.1G1 cells derived from a LigI-deficient patient or in human cells depleted in LigI by shRNA targeting. **a** Detection of endogenous LigIII, XRCC1 or LigI (in *red*) and BrdU incorporation sites (*green*) in AS3WT2 human cells derived from a healthy donor and 46Br.1G1 cells obtained from a LigI-deficient patient. Co-localization of the LigIII, XRCC1 or LigI proteins and the BrdU-positive replication foci appears as *yellow spots* in the merged images. **b** Endogenous LigIII (*red*) and PCNA foci (*green*) in control HeLa cells (HeLa^CTL^), LigI-depleted HeLa cells (HeLa LigI^KD^) or LigIII-depleted HeLa cells (HeLa LigIII^KD^). The co-localization of LigIII protein and PCNA foci appears as *yellow spots* in the merged images. *Scale bar* on all merged images, 5 μm. **c** Thresholded merge images were used to determine the percentage of nuclei from AS3WT2 and 46Br.1G1 cells lines (*left panel*) and from HeLa^CTL^ and HeLa LigI^KD^ cell lines (*right panel*) with more than two typical mid- or late-replication structures are presented for the indicated co-staining

Comparison of the human fibroblastic line 46Br.1G1 derived from a patient with a LigI-deficiency syndrome with a control AS3WT2 line derived from a healthy donor also demonstrated a significant increase in the co-localization of either LigIII or XRCC1 with either BrdU (Fig. 2a) or PCNA foci (ESM Fig. 2). The percentage of cells with mid- and late-S-phase replication foci co-stained for LigIII/BrdU or LigIII/PCNA was, respectively, 22 and 27.5 % in AS3WT2 cell line compared to 88.5 and 87.5 % in 46Br.1G1 cell line (Fig. 2c and ESM Fig. 2), and the percentage of nuclei with co-stained for XRCC1/BrdU S phase replication foci was 25.5 % in the AS3WT2 cell line and 69 % in 46Br.1G1 cell line (Fig. 2c).

**Fig. 3:**
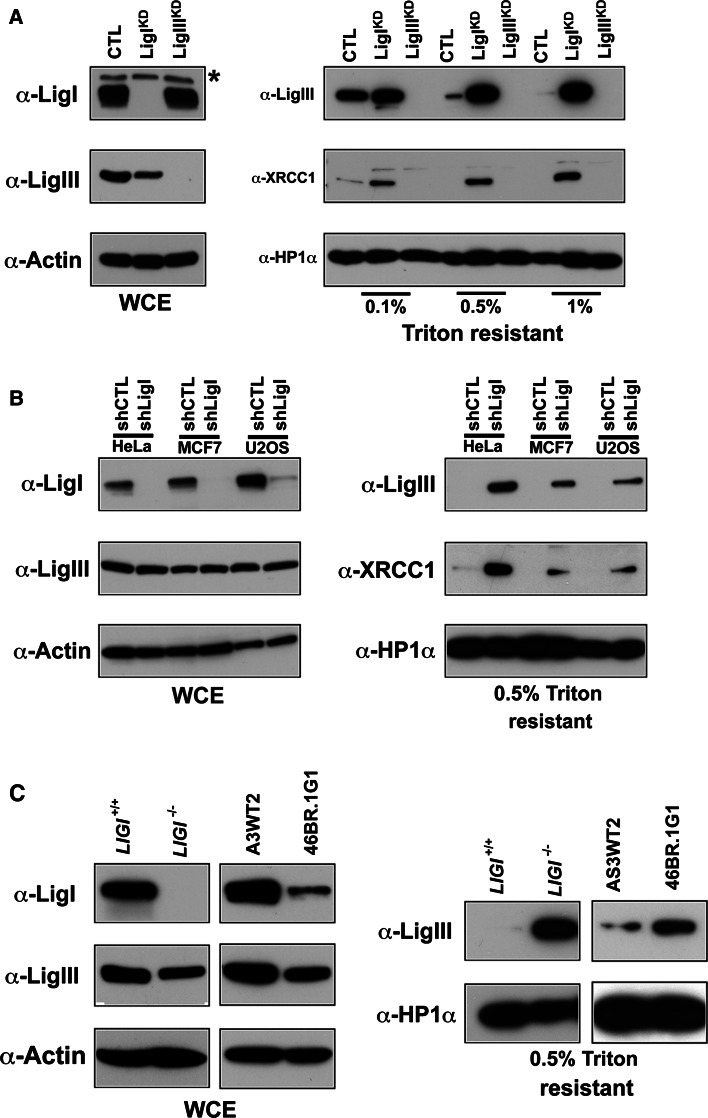
Increase in chromatin-bound LigIII and XRCC1 in LigI-depleted cells. **a** Asynchronously growing HeLa cell populations expressing control, *LIGI*, or *LIGIII* shRNAs from the pEBV episomal plasmid were harvested and divided into two fractions: whole cell extracts (WCE) and extracts prepared with the indicated Triton concentrations to yield the insoluble fraction. The WCE preparation (*left panel*) and Triton-resistant extracts (*right panel*) were then analyzed by Western blotting with the indicated antibodies. *Non-specific band. **b** WCE (*left panel*) and the 0.5 % Triton-resistant extracts (*right panel*) were also prepared from lentiviral-transduced HeLa, MCF7, and U2OS cell populations expressing the indicated shRNA and then analyzed by Western blotting with the indicated antibodies. **c** WCE (*left panel*) and 0.5 % Triton-resistant (*right panel*) *LIGI* ^+/+^ and *LIGI* ^−/−^ mouse cell line and AS3WT2 and 46Br.1G1 human cell line extracts analyzed by Western blotting with the indicated antibodies

In order to analyze LigI depletion in the same genetic background, we used HeLa cells to target LigI or LigIII expression by introducing shRNA vectors. We transfected an episomal plasmid to generate a HeLa^CTL^ control cell line expressing a control shRNA, and HeLa LigI^KD^ and LigIII^KD^ line expressing an shRNA targeting *LIGI* or *LIGIII*, respectively [34]. Western blots are presented in Fig. 3b. We observed that in the HeLa LigI^KD^ cells, LigIII immunostaining was mostly diffuse with distinct and intense foci in mid- and late-S-phase cells, while no LigIII staining was observed in the HeLa LigIII^KD^ cells (Fig. 2b). Depletion of the LigI protein in proliferating HeLa LigI^KD^ cells was found to be associated with an increase in the percentage of nuclei with co-stained for LigIII/PCNA S phase replication foci from 25.5 % in the HeLa^CTL^ cell line to 61 % in HeLa LigI^KD^ cell line (Fig. 2b, c). Taken together, these observations show that the accumulation of the LigIII and XRCC1 proteins to BrdU or PCNA foci in proliferating cells is a conserved response to a LigI deficiency between mouse and human cell lines.

### DNA ligase III and XRCC1 proteins are recruited to chromatin in DNA ligase I-deficient cells

It has been shown that the LigIII/XRCC1 complexes localized at DNA damage sites are resistant to detergent extraction [47–49]. To assess if the XRCC1 and LigIII proteins were tightly bound to the nuclear fraction in proliferating LigI-deficient cell lines, we treated HeLa^CTL^ and HeLa LigI^KD^ cell lines with Triton X-100-containing buffers to extract the soluble fraction and analyzed the insoluble chromatin and/or nuclear matrix fraction for its LigIII and XRCC1 content by Western blotting. We found that the LigIII and XRCC1 proteins were efficiently extracted from HeLa^CTL^ cells treated with 0.5 and 1 % Triton containing buffer. In contrast, under the same extraction conditions, the LigIII and XRCC1 proteins were retained in the Triton-resistant fraction from HeLa LigI^KD^ cells (Fig. 3a). We next analyzed the solubility of the LigIII and XRCC1 proteins from HeLa, MCF7, and U2OS cells in which an anti-*LIGI* shRNA was introduced by lentiviral transduction. Less than 1 % of the total amount of LigIII or XRCC1 could be detected in the 0.5 % Triton-resistant fraction from control populations in any of the three cell types. However, a significant amount of LigIII and XRCC1 proteins were found to be resistant to detergent extraction in the LigI-depleted populations (Fig. 3b). Both 46Br.1G1 cells and *LIGI* ^−/−^ mouse cell lines have a lower level of LigIII protein, however, more LigIII protein was detected in the insoluble fraction from these cells than in the respective control lines (Fig. 3c). In conjunction with the observation of the co-localization of the LigIII and XRCC1 proteins at replication foci, these results showing a tight binding of LigIII and XRCC1 to the chromatin suggest a role for the LigIII/XRCC1 complex in the replication processes in LigI-deficient cells.

### The viability of LigI-deficient cells is dependent on the LigIII, XRCC1, and PARP-1 proteins

**Fig. 4:**
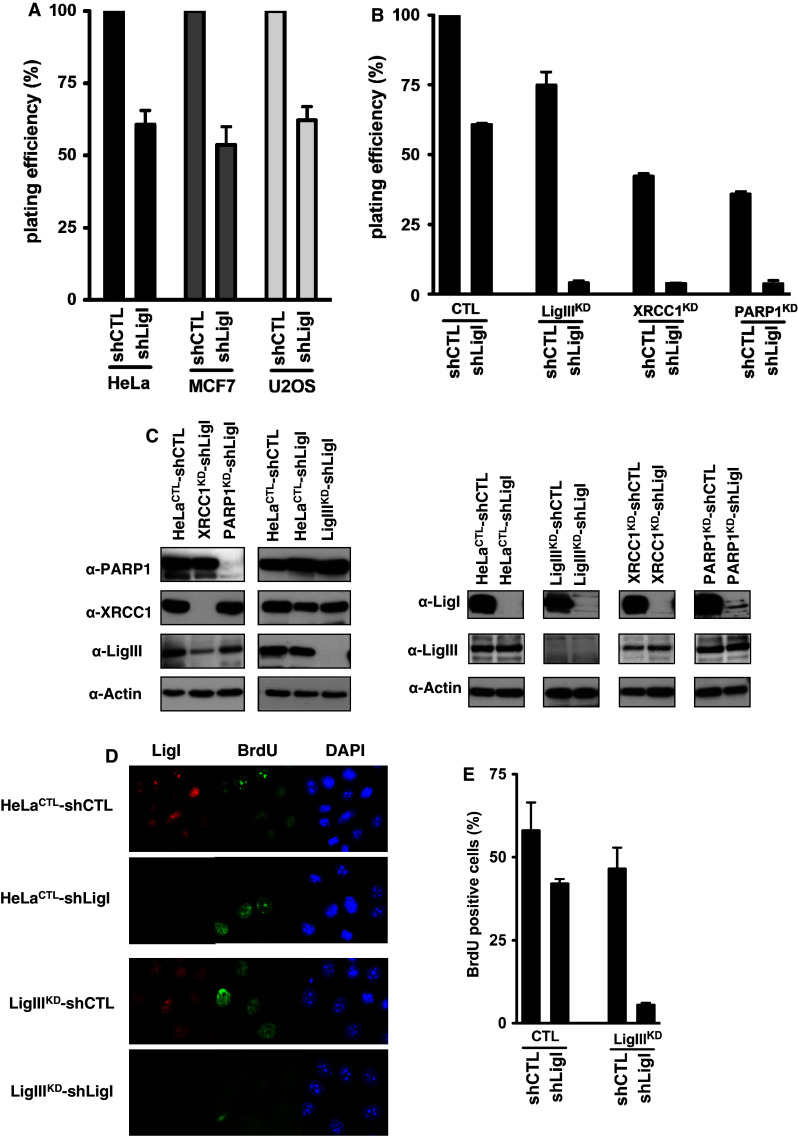
LigIII is required for the viability and efficient DNA replication of the LigI-deficient cells. **a** Effects of LigI-depletion on the plating efficiency in HeLa, MCF7, and U2OS cells following lentiviral transduction with a *LIGI* shRNA (shLigI) were compared to the respective control (shCTL) cell populations. **b** The control shRNA (shCTL) or *LIGI* shRNA (shLigI) molecules were introduced by lentiviral transduction of HeLa cells expressing either a control (CTL), *LIGIII* (LigIII^KD^), *XRCC1* (XRCC1^KD^), or *PARP1* (PARP1^KD^) shRNA from the pEBV episomal plasmid. The plating efficiency of the cell populations expressing the indicated pair of shRNAs was compared to that of the HeLa^CTL^-shCTL cells expressing the control shRNAs. **c** The LigI, LigIII, XRCC1, and PARP1 protein levels were analyzed by Western blotting. **d** Two days post-infection, LigI-depletion was verified at the cellular level by immunofluorescence (*red*) and the replicating cells were detected by BrdU incorporation (*green*). **e** Percentages of BrdU-positive cells detected in the indicated HeLa cells populations following a 30-min BrdU pulse. The values ± SD represent the mean of two experiments for which at least 150 nuclei per experiment were scored

We next determined the effect of LigI depletion on the plating efficiencies of HeLa, MCF7, and U2OS cells expressing *LIGI* shRNA via lentiviral transduction. The resulting HeLa shLigI, MCF7 shLigI, and U2OS shLigI cells showed reduced colony formation to 60, 54, and 62 % of the levels found for the respective control shRNA cells (Fig. 4a, Western blots are presented in Fig. 3b). To investigate the requirements for LigIII and XRCC1 in colony formation in the context of a LigI defects in HeLa cells, we targeted these proteins by expressing shRNAs (Fig. 4c). Because of the role of the SSB DNA sensor PARP-1 protein during DNA strand repair, the function of PARP-1 in colony formation in the context of a LigI defect in HeLa cells was also analyzed. The plating efficiencies of the HeLa LigIII^KD^, HeLa XRCC1^KD^, and HeLa PARP-1^KD^ cell populations were 75, 42, and 35 %, respectively, compared to HeLa^CTL^ control cells (Fig. 4b). The additional depletion of LigI in these cells by the introduction of a *LIGI* targeting shRNA via lentiviral transduction reduced the plating efficiency of all the populations to less than 4 % of the controls at 15 days post-infection (Fig. 4b). In addition to confirming the requirement of the PARP-1 for the viability of LigI-deficient cells [5, 25, 50], these results show that the repair proteins LigIII and XRCC1 are also required for the viability of LigI-impaired cells.

Furthermore, we compared the DNA replication efficiency by a BrdU incorporation assay 2 days post-depletion in single-DNA ligase depleted cell populations (HeLa LigIII^KD^ and HeLa CTL-shLigI) and double-DNA ligase depleted cell populations (HeLa LigIII^KD^-shLigI) (Fig. 4c, d, e). The percentages of BrdU-positive cells were similar in the single-LigI and LigIII-depleted populations (42 % in HeLa CTL-shLigI compared to 46.5 % in HeLa LigIII^KD^-shLigI). However, the percentage of BrdU-positive cells was dramatically reduced in HeLa LigIII^KD^-shLigI cells (5.5 %). These results suggest that the reduced viability of cells depleted for both LigI and LigIII results from a defect in the replication process.

### LigI and LigIII cooperate to prevent SCEs

**Fig. 5:**
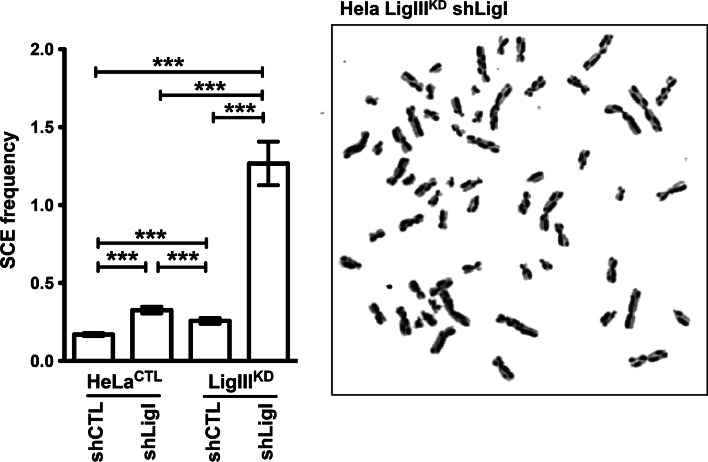
LigI and LigIII cooperate in the suppression of SCEs. A representative image of metaphase spreads prepared for SCE analysis from HeLa cells depleted in both LigI and LigIII. The histogram on the *left* shows the average SCE frequency per chromosome in the different cell types calculated from 37 to 50 metaphases for each cell lines ***p < 0.0001

One of the hallmarks of 46Br.1G1 cells [5, 6] and LigIII deficiency in mouse cells [7] is the increase in the incidence of SCEs. The percentages of SCEs per chromosome (SCE frequency) in metaphase spreads from HeLa, MCF7, and U2OS cells transduced with an *LIGI* shRNA were determined. An increase in the SCE frequency of 1.9-, 1.5-, and 1.4-fold, respectively, (*p* < 0.0001) in the HeLa, MCF7, and U2OS LigI-depleted cells compared to their corresponding controls (Table 1) was found. The LigIII protein expression level was not affected upon LigI depletion in these cell lines (Fig. 3b). To analyze the impact of both LigI and LigIII depletion on the SCE frequency, HeLa^CTL^ or HeLa LigIII^KD^ cell lines were transduced either with a control or *LIGI* targeting shRNA-producing HeLa^CTL^-shCTL, HeLa LigIII^KD^-shCTL, HeLa^CTL^-shLigI and HeLa LigIII^KD^-shLigI cell populations. A similar XRCC1 protein level was observed in HeLa cells depleted for LigI or LigIII or in both LigI and LigIII-depleted cells (Fig. 4c). The SCE frequencies in these cells were then measured at 3 days post-transduction, an early time point where a fraction of HeLa LigIII^KD^-shLigI cells was still dividing. When compared to HeLa^CTL^-shCTL cells, the SCE frequencies in the HeLa^CTL^-shLigI cells and HeLa LigIII^KD^-shCTL cells were increased by 1.9-fold (*p* < 0.0001) and 1.5-fold, respectively (*p* < 0.0001) (Fig. 5). The observed SCE frequencies in the HeLa^CTL^-shLigI (0.326 % SCEs) and HeLa LigIII^KD^-shCTL (0.257 % SCEs) cells were also significantly different (*p* < 0.0001) (Fig. 5). In cells depleted for both LigIII and LigI (HeLa LigIII^KD^-shLigI) cells, the SCE frequency was 1.26 %, which is a striking increase compared to the HeLa^CTL^-shCTL cells (7.5-fold, *p* < 0.0001), HeLa^CTL^-shLigI cells (5.2-fold, *p* < 0.0001) or the HeLa LigIII^KD^-shCTL cells (4.8-fold, *p* < 0.0001) (Fig. 5). Based on this observed synergistic increase in SCEs in the LigI–LigIII-depleted cells, we speculate that LigI and LigIII can complement each other in the suppression of SCEs.

**Table 1:** The SCE frequency increases in cells depleted of LigI by lentiviral transduction

Cell line	SCE^a^	*p* ^b^	*n* ^c^
HeLa shCTL	0.169 ± 0.010		43 (2,839)
HeLa shLigI	0.326 ± 0.021	<0.0001	51 (3,370)
MCF7 shCTL	0.194 ± 0.009		40 (2,739)
MCF7 shLigI	0.281 ± 0.016	<0.0001	40 (2,728)
U2OS shCTL	0.141 ± 0.008		43 (3,184)
U2OS shLigI	0.216 ± 0.042	<0.0001	38 (2,776)

^a^Equals the total number of sister chromatid exchanges divided by the total number of chromosomes counted; ± indicates the 95 % confidence interval

^b^Determined via the Mann–Whitney test

^c^Number of metaphase spreads from which the SCE frequency was determined; the* values in parentheses* indicate the total number of chromosomes analyzed in each lentiviral-transduced cell population

### The depletion of LigI but not LigIII induces sister telomere fusion

**Fig. 6:**
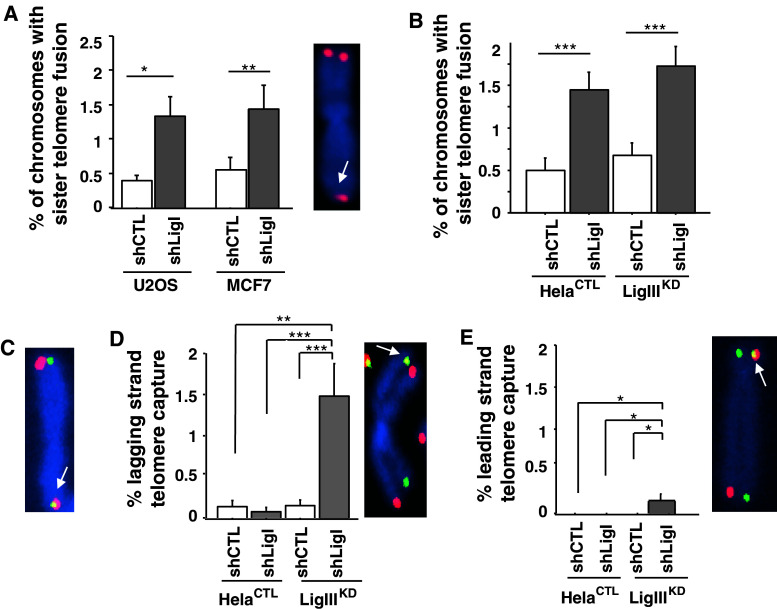
Telomere instability in LigI-depleted HeLa cells. **a** Percentages (mean ± SD) of sister telomere fusions per chromosome determined from Telo-FISH-stained metaphase spreads of the indicated transduced cells and an example of a chromosome with a sister telomere fusion (*white arrow*). **b** Percentages of chromosomes with sister telomere fusion determined from Telo-FISH-stained metaphase spreads for the indicated cell populations. **c** Example of sister telomere fusion (*yellow, white arrow*) involving both leading (*green*) and lagging (*red*) strand telomeres. **d** Percentage of chromosomes with lagging strand telomere capture in the different cells indicated (*left*) and example of lagging strand telomere capture detected by CO-FISH (*white arrow*). **e** Percentage of chromosomes with leading strand telomere capture in the different cells indicated (*left*) and example of leading strand telomere capture detected by CO-FISH (*white arrow*). Percentages were calculated from 32–42 metaphases for each cell line. Statistical significance levels are noted as follows: ***p < 0.0005, **p < 0.005, and *p < 0.05

The consequences of LigI depletion on telomere stability were next investigated by Telo-FISH staining of chromosomes from the telomerase-positive cell line MCF7 and the U2OS cell line. In both lines, an increase in the percentage of chromosomes with sister telomere fusions was observed when LigI was depleted by lentiviral transduction (0.3 % in U2OS shCTL and 1.2 % in U2OS shLigI cells, *p* = 0.002; 0.5 % in MCF7 shCTL, and 1.4 % in MCF7 shLigI cells, *p* = 0.03; Fig. 6a). We also compared these rates in HeLa cells depleted in LigIII, LigI, or both DNA ligases. A sister telomere fusion rate of 1.4 % in the HeLa^CTL^-shLigI cells, which represents a 2.8-fold (*p* = 0.004) and twofold (*p* = 0.004) increase compared to HeLa^CTL^-shCTL and HeLa LigIII^KD^-shCTL cells, respectively, was observed (Fig. 6b). The percentages of chromosomes with sister telomere fusions in HeLa LigIII^KD^-shCTL and HeLa^CTL^-shCTL cells were not significantly different (*p* = 0.4). However, 1.7 % of chromosomes exhibited sister telomere fusions in the HeLa LigIII^KD^-shLigI cells depleted for both ligases, which is a significant increase compared with HeLa^CTL^-shCTL (*p* < 0.0001) and HeLa LigIII^KD^-shCTL (*p* < 0.002) cells. Interestingly, the HeLa^CTL^-shLigI and the HeLa LigIII^KD^-shLigI cells showed a similar rate of sister telomere fusions (*p* = 0.4). Hence, a defect in LigI but not in LigIII was found to be associated with an increased occurrence of sister telomere fusions.

We next performed CO-FISH, which allows the identification of newly replicated lagging (stained in red) and leading (stained in green) strand telomeres of metaphasic chromosomes [39]. This analysis confirmed the presence of both lagging and leading strand telomeres at fused sister chromatids in the HeLa^CTL^-shLigI and the HeLa LigIII^KD^-shLigI cells (Fig. 6c). Chromosome orientation-FISH did not, however, reveal the induction of reciprocal sister telomere exchange (T-SCE) in HeLa cells depleted of LigI or LigIII, either individually or together. In contrast, we found a significant increase in non-reciprocal telomeric strand exchanges in HeLa LigIII^KD^-shLigI cells (1.8 % of chromosomes vs. less than 0.2 % in the other cell types tested). Non-reciprocal telomeric strand exchanges were detected either by the presence of leading strand telomere DNA in the lagging strand telomeres (referred to as leading strand telomere capture; Fig. 6d) or by the presence of telomere lagging strand DNA in the leading strand telomeres (referred to as lagging strand telomere capture; Fig. 6e) resulting in yellow staining at the telomeres. Interestingly, the HeLa LigIII^KD^-shLigI cells exhibited the most lagging strand telomere captures. Taken together, these data suggest that LigI defects induce telomere instability as a consequence of DNA breaks in the lagging strand telomeres, which result in subsequent sister telomere fusion, and are not prevented by LigIII. However, the lagging strand telomere captures in HeLa LigIII^KD^-shLigI cells are likely to be the consequence of additional breaks within lagging strand telomeric DNA. Hence, their absence in HeLa^CTL^-shLigI cells, in contrast to HeLa LigIII^KD^-shLigI cells, indicates that LigIII partially compensates for the LigI defect and thereby reduces some of the lagging strand telomere instability.

### The depletion of LigI increases the incidence of mitotic abnormalities

**Fig. 7:**
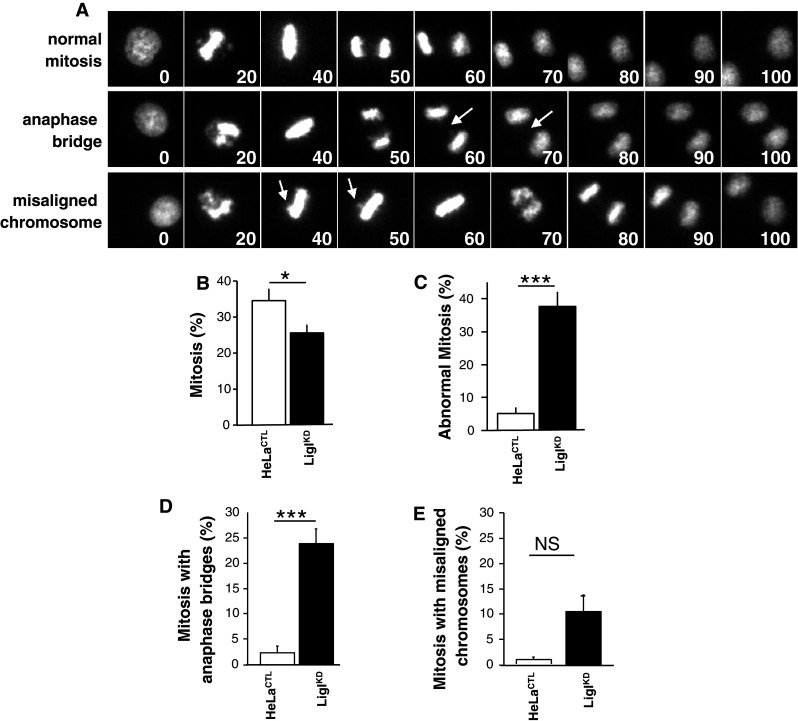
LigI-defect promotes an increased number of mitotic abnormalities. **a** Representative time-lapse images of HeLa H2B-GFP LigI^KD^ mitosis. The videos of these mitotic events were synchronized from the onset of prophase. The* numbers* indicate the time in minutes, the *arrows* point to bridged DNA (*middle row*) and misaligned chromosomes (*lower row*). **b** Percentage of mitoses in control (*open bar*) and LigI^KD^ HeLa H2B-GFP (*closed bar*). **c** Percentage of abnormal mitoses in control and LigI^KD^ HeLa H2B-GFP. **d** Percentage of abnormal mitoses with anaphase bridges. **e** Percentage of abnormal mitoses with misaligned chromosomes. A total of 257 and 270 mitoses were analyzed, respectively. Statistical significance levels are noted as follows:* NS* not significant, *p < 0.02 and (***p < 0.0001)

We next followed the progression of mitosis in HeLa H2B-GFP^CTL^ and HeLa H2B-GFP LigI^KD^ cells through live microscopy (Fig. 7a, b and ESM video 1, 2 and 3). The depletion of LigI in HeLa H2B-GFP cells via an episomal plasmid dramatically increased the incidence of abnormal mitoses (37.6 % of the mitoses in HeLa H2B-GFP LigI^KD^ cells compared to only 5 % in the HeLa H2B-GFP^CTL^ control cells; *p* = 0.0001) (Fig. 7c). These defects were mainly due to anaphase bridges (23.8 % of mitoses in HeLa H2B-GFP LigI^KD^ cells compared to 2.3 % in HeLa H2B-GFP^CTL^; *p* = 0.0001) (Fig. 7d and ESM video 2) and misaligned chromosomes (10.5 % of mitoses in HeLa H2B-GFP LigI^KD^ compared with 0.8 % in HeLa H2B-GFP^CTL^; *p* = 0.08) (Fig. 7e and ESM video 3). These mitotic abnormalities through the onset of chromosome misalignments and anaphase bridges were most probably initiated as the cells progressed through mitosis with sister telomere fusions resulting from the LigI defect. Hence, our results suggest that a reduction in viability of LigI-deficient cells is at least partially due to sister telomere fusions resulting from lagging strand telomere instability and therefore that LigIII cannot functionally compensate for the loss of LigI in this regard.

## Discussion

We have shown here that in mammalian LigI-deficient cells, LigIII and XRCC1 are retained on chromatin at replication foci and that both proteins are required for the long-term survival of these cells. We further show for the first time that both LigI and LigIII cooperate to suppress SCEs, but that LigIII is not sufficient to prevent sister telomere fusions in LigI-deficient cells, revealing the importance of LigI function in telomere stability.

The proliferation of mouse embryonic fibroblast cell lines derived from *LIGI* ^−/−^ animals [5, 6, 26, 30] indicates that alternative pathways can compensate for this defect and contribute to cell viability. LigaseIII, like LigI, catalyze the sealing of SSBs. LigaseIII recruitment during DNA repair onto a ligatable substrate is dependent on its SSB-binding domain [51] and/or on the nick- and gap-DNA-binding protein XRCC1 [52, 53]. We have shown that LigIII and XRCC1 proteins co-localize with replication sites and become Triton-resistant when LigI function is compromised (Figs. 1, 2, 3), thus resembling the LigIII/XRCC1 foci induced by DNA damage [47, 48, 54–56]. Moreover, the viability of LigI-deficient human cells is dependent on LigIII, XRCC1, and PARP-1 and both LigI and LigIII are required for efficient DNA replication (Fig. 4). These observations are consistent with a recent study reporting that in chicken cells (DT40) LigIII can efficiently substitute for all major DNA replication functions of LigI [57]. We have also shown that PARP-1 is required to support the viability of LigI-deficient cells, which is consistent with previous studies showing that PARP activity inhibition is deleterious in conjunction with a LigI defect [5, 25, 50]. Hence, the LigIII/XRCC1 foci formation during S phase in LigI-deficient cells is likely to be dependent on PARP-1 activity, which is known to facilitate the recruitment of LigIII/XRCC1 complex to single-strand interruptions [10, 58].

The detection of the LigIII and the XRCC1 proteins at/or close to the replication sites and their requirement for the viability of LigI-deficient cells strongly suggest that the XRCC1/LigIII complex contributes to the sealing of lagging strand replication intermediates behind replication forks in LigI-deficient cells. Previous investigations of the consequences of a LigI defect have led to conflicting results. It has been reported that perturbing the function of the human LigI orthologue in yeast [59], plants [60], chicken B cell line [57], and mice [26] results in S phase progression defects compared to their wild-type counterparts. In contrast, other studies observed a normal cell cycle distribution in mice and human LigI-deficient cells [5, 61]. The 46Br.1G1 cells, which have low LigI activity, can proliferate despite persisting DNA damage. Soza and colleagues have suggested that an adaptive response may underlie the proliferation of these cells [61]. The maturation of newly synthesized Okazaki fragments is delayed in the 46Br.1G1 cell line and in the *LIGI* ^−/−^ mouse embryonic fibroblast cell lines [5, 6, 26, 27]. It has been thus proposed that another DNA ligase may partially compensate for the LigI defect in these LigI-deficient proliferating cells [30, 49]. We have shown that the LigIII/XRCC1 proteins are present at/or close to the replication sites in cells with impaired LigI function (Figs. 1, 2). The replication defect observed in LigI–LigIII-depleted cells suggests that the proliferation of LigI-deficient cells may therefore rely on the recruitment of the LigIII/XRCC1 complex. Of note in this regard, it should be pointed out that there is no DNA ligase III homolog in yeast and plants, which could explain why LigI depletion severely affects S phase progression in these organisms (for review, see [15]).

The interaction of XRCC1 with replication factors [62–65] and its requirement for a replication fork restart post-MMS (methyl methanesulfonate) treatment [35, 66–68] has led to the hypothesis that XRCC1 may be involved in the coordination of replication-coupled repair. It is therefore likely that XRCC1, in association with LigIII, could favor the joining of unligated replication intermediates in LigI-deficient cells, which is also likely to require PARP-1 [69, 70] possibly through its role in the regulation of replication fork progression [71]. The replication-coupled SSBR pathway involving XRCC1 and a number of its known binding proteins [49, 62, 63, 65] may promote the joining of SSBs during replication fork progression in LigI-deficient cells, thus allowing S phase progression.

Both the small increase in the spontaneous SCE frequency in LigI- or in LigIII-deficient cells, observed here (Fig. 5) and previously by others [5–7], and the dramatic synergistic increase in the frequencies of spontaneous SCEs in the LigI–LigIII-depleted cells, show that LigI and LigIII can efficiently compensate for each other to suppress SCE. Hence, our present observations support a model in which the ligation of replication intermediates in LigI-deficient cells is dependent on the LigIII, albeit with a delay compared to LigI-proficient cells [5, 6, 26, 27]. This LigI and LigIII functional redundancy would serve to prevent the formation of DSBs as the replication forks progresses towards SSBs, which can otherwise result in the loss of cell viability and in the formation of SCEs [72]. Partial redundancy between the LigI and LigIII during DNA repair was shown in vivo and in vitro [73, 74]; it was observed that in LigIII-deficient mice, the LigI is involved in translocation formation by alternative NHEJ [20–22] and previous studies that have analyzed ligation reactions using in vitro DNA repair assays have shown a partial overlap in the functions of LigI and LigIII [73, 74].

We further show in our current experiments that LigIII could not totally prevent telomere instability resulting from a LigI defect. A defect in lagging-strand processing proteins including polymerase α, the endonuclease FEN-1 and the helicase/nuclease Dna2 is associated with compromised telomere functions [75–78]. In mouse cells, a polymerase-α defect results in the elongation of single-stranded telomeric G-strand overhangs and in Robertsonian translocations [75], whereas an FEN-1 defect induces telomere shortening [77] and telomere end-to-end fusions [76, 77]. To our knowledge, our present study is the first report showing that a LigI defect induces sister telomere fusions (Fig. 6).

Both leading- and lagging-strand telomeres are protected from end-to-end fusions by a specific telomeric nucleoprotein complex and a DNA architecture that includes a 3′-overhang. Events known to render sister chromatids prone to fusion by NHEJ after replication [79, 80] include altered sister chromatid dissociation [79] or impaired formation of the telomere protective conformation [32, 40, 41]. The remaining SSBs resulting from compromised Okazaki fragment ligation during telomere replication in LigI-deficient cells could render the lagging-strand telomere more prone to DSBs. Broken lagging-strand telomeres will be fused by NHEJ with the newly replicated leading chromatid at which the protective G-strand overhang has not yet been formed [81, 82] and give rise to the dramatic increase in sister telomere fusions observed in LigI-deficient cells. We cannot exclude from our data the possibility that telomere breakage resulting from impaired SSB repair in LigI-deficient cells can also contribute to sister telomere fusions.

Although we have demonstrated here that LigIII does not compensate for the function of LigI that prevents sister telomere fusions, the low frequency of lagging- or leading-strand telomere capture in LigI-deficient and LigIII-deficient cells compared with LigI-LigIII-deficient cells clearly demonstrates that both LigI and LigIII can participate in the maintenance of telomere stability by preventing telomeric strand capture. Lagging-strand telomere capture in HeLa LigIII^KD^-shLigI cells is likely the consequence of additional breaks within lagging strand telomeric DNA. Hence, their absence in HeLa^CTL^-shLigI cells, in contrast to HeLa LigIII^KD^-shLigI cells, indicates that LigIII at least partially compensates for the LigI defect, thus reducing if not preventing lagging-strand telomere instability. These observations are consistent with the role of DNA repair proteins including PARP-1 and the polymerase-β protein in the maintenance of telomere integrity [83, 84].

In conclusion, our present observations show that the LigIII/XRCC1 complex is required to promote the viability of mammalian LigI-deficient cells and that LigI and LigIII efficiently cooperate during DNA replication and to inhibit a high frequency of SCE events. However, LigIII cannot fully compensate for the loss of LigI function at telomeres, which results in lagging-strand telomere instability and sister telomere fusions. This reveals the importance of LigI for telomere stability.

### Electronic supplementary material

Below is the link to the electronic supplementary material.

Supplementary Fig 1 **Fig 1 Analysis of the co-localization of LigIII and XRCC1 with DNA replication foci in wild-type (** ***LIGI*** ^**+/+**^ **) and** ***LIGI*** **knockdown (** ***LIGI*** ^**-/-**^ **) mouse cells**. (a) Endogenous LigIII (red) was co-immunodetected with BrdU incorporation sites or PCNA staining (green) in early-S phase *LIGI* ^+/+^ and *LIGI* ^-/-^ mouse cells. (b) Endogenous XRCC1 (red) was co-immunodetected with the BrdU incorporation site (green) or (c) with PCNA staining (green). Overlaps between XRCC1 and BrdU or PCNA signals were observed in late-S phase *LIGI* ^-/-^ cells. The thresholded images were obtained as described in Materials and Methods. For each double staining combination, more than 40 nuclei were analyzed per cell line. The percentages are presented in Figure 1c. Scale bar: 5 μm (PPT 9020 kb).

Supplementary Fig 2 **Fig 2** (a) Immunodetection of endogenous LigIII (red) and PCNA foci (green) in late-S phase control AS3WT2 cells or in late-S phase 46Br.1G1 cells derived from a LigI-deficient patient. The co-localization of LigIII protein and PCNA foci appears as yellow spots in the merged images. Scale bar: 5 μm. (b) Percentages of nuclei from AS3WT2 and 46Br.1G1 cell lines with more than two late-replication structures (ring-shape or horseshoe staining) for the PCNA/LigIII co-staining (PPT 4313 kb).

Supplementary material 3 (AVI 1141 kb). **Video 1** Time-lapse video microscopy of a normal HeLa H2B-GFP^CTL^ cell mitosis

Supplementary material 4 (AVI 1141 kb). **Video 2** Time-lapse video microscopy showing an anaphase bridge during mitosis in HeLa H2B-GFP LigI^KD^ cell

Supplementary material 5 (AVI 1141 kb). **Video 3** Time-lapse video microscopy showing chromosome misalignment during mitosis in HeLa H2B-GFP LigI^KD^cell

## Abbreviations

SSB: Single-strand break
DSB: Double-strand break
NHEJ: Non-homologous end-joining
SCE: Sister chromatid exchange
Telo-FISH: Telomere-fluorescent in situ hybridization
CO-FISH: Chromosome orientation-FISH
