# Supplementary material for: Partial complementation of a DNA ligase I deficiency by DNA ligase III and its impact on cell survival and telomere stability in mammalian cells
Source: Cell Mol Life Sci. 2012 Mar 30;69(17):2933–49. doi: 10.1007/s00018-012-0975-8 (PMC3417097; doi:10.1007/s00018-012-0975-8)
Supplement: Supplementary file 1 — Supplementary Fig 1 Fig 1 Analysis of the co-localization of LigIII and XRCC1 with DNA replication foci in wild-type ( LIGI +/+ ) and LIGI knockdown ( LIGI -/- ) mouse cells. (a) Endogenous LigIII (red) was co-immunodetected with BrdU incorporation sites or PCNA staining (green) in early-S phase LIGI +/+ and LIGI -/- mouse cells. (b) Endogenous XRCC1 (red) was co-immunodetected with the BrdU incorporation site (green) or (c) with PCNA staining (green). Overlaps between XRCC1 and BrdU or PCNA signals were observed in late-S phase LIGI -/- cells. The thresholded images were obtained as described in Materials and Methods. For each double staining combination, more than 40 nuclei were analyzed per cell line. The percentages are presented in Figure 1c. Scale bar: 5 μm (PPT 9020 kb). [file 18_2012_975_MOESM1_ESM.ppt]

## Slide 1
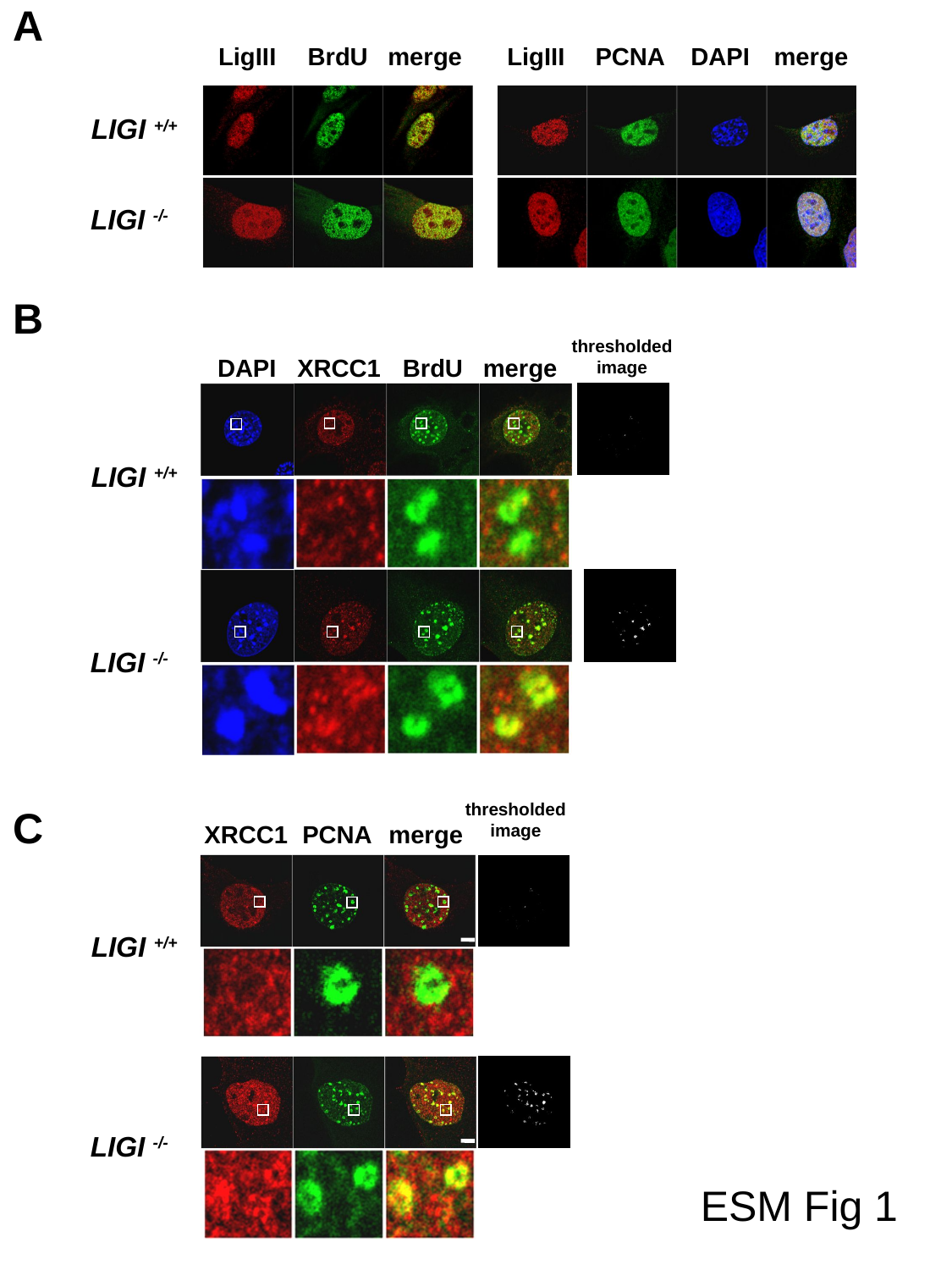

A
LigIII
BrdU
merge
LigIII
PCNA
DAPI
merge
LIGI +/+
LIGI -/-
B
thresholded
image
DAPI
XRCC1
BrdU
merge
LIGI +/+
LIGI -/-
thresholded
image
C
XRCC1
PCNA
merge
LIGI +/+
LIGI -/-
ESM Fig 1
