# Supplementary figures and images for: Partial complementation of a DNA ligase I deficiency by DNA ligase III and its impact on cell survival and telomere stability in mammalian cells
Source: Cell Mol Life Sci. 2012 Mar 30;69(17):2933–49. doi: 10.1007/s00018-012-0975-8 (PMC3417097; doi:10.1007/s00018-012-0975-8)

## Slide 1
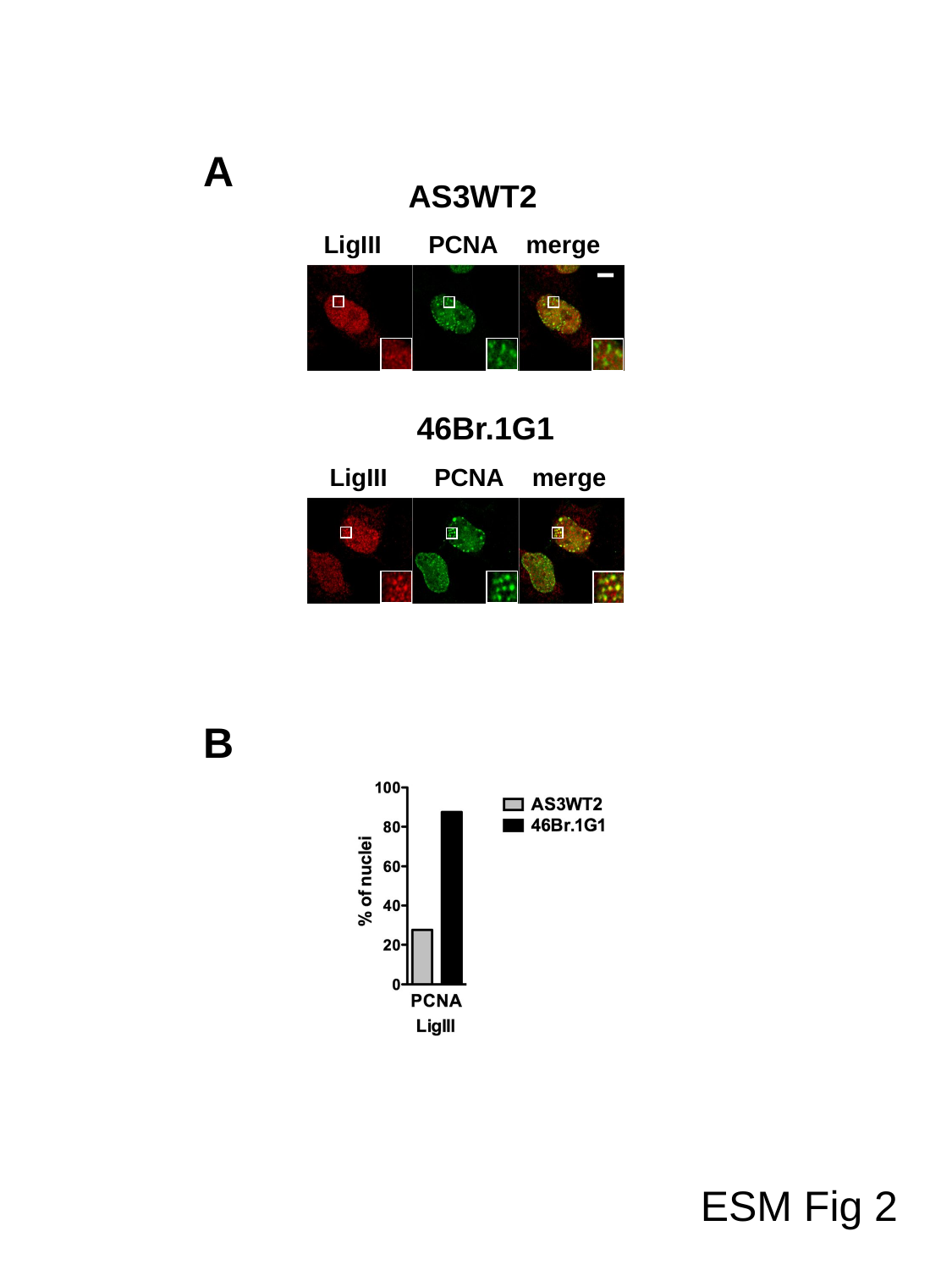

A
AS3WT2
LigIII
PCNA
merge
46Br.1G1
LigIII
PCNA
merge
B
ESM Fig 2

Supplement: Supplementary file 2 — Supplementary Fig 2 Fig 2 (a) Immunodetection of endogenous LigIII (red) and PCNA foci (green) in late-S phase control AS3WT2 cells or in late-S phase 46Br.1G1 cells derived from a LigI-deficient patient. The co-localization of LigIII protein and PCNA foci appears as yellow spots in the merged images. Scale bar: 5 μm. (b) Percentages of nuclei from AS3WT2 and 46Br.1G1 cell lines with more than two late-replication structures (ring-shape or horseshoe staining) for the PCNA/LigIII co-staining (PPT 4313 kb). [file 18_2012_975_MOESM2_ESM.ppt]
